# A Case of Marine-Lenhart Syndrome with a Negative TSH Receptor Antibody Titer Successfully Treated with a Fixed, Low Dose of I^**131**^

**DOI:** 10.1155/2014/423563

**Published:** 2014-08-03

**Authors:** Masahiro Takei, Hiroaki Ishii, Yoshihiko Sato, Mitsuhisa Komatsu

**Affiliations:** Division of Diabetes, Endocrinology and Metabolism, Department of Internal Medicine, Shinshu University School of Medicine, Matsumoto, Nagano 390-8621, Japan

## Abstract

We herein describe a case of Marine-Lenhart syndrome with a negative TSH receptor antibody titer. A 75-year-old female presented to our hospital with malaise, palpitations, and mild fine tremors. She did not have any signs suggestive of Graves' ophthalmopathy, including conjunctival injection, periorbital edema, or proptosis. Her laboratory data were negative for thyroid autoantibodies, including anti-thyroid peroxidase antibodies, anti-thyroglobulin antibodies, and anti-TSH receptor antibodies (TRAb). Ultrasonography of the thyroid gland revealed a tumor in the right lobe. The remaining thyroid gland had an inhomogeneous and rough texture with a high color Doppler flow. I^123^ scintigraphy disclosed a hot nodule in the right thyroid gland corresponding to the tumor detected on ultrasonography, suggesting Plummer disease. Furthermore, there was an increased uptake of radionuclide in the rest of the thyroid gland, despite the suppressed level of TSH and negative titer of TRAb, suggesting underlying Graves' disease. The present findings suggested a diagnosis of Marine-Lenhart syndrome with a negative TRAb titer. Treatment with 10 mCi of radioiodine was highly effective in treating hyperthyroidism in this case. A negative TSH receptor antibody titer does not necessarily rule out the existence of Graves' disease in patients with Plummer disease.

## 1. Introduction

Thyrotoxicosis results from autonomic thyroid hyperfunction or destructive processes [1]. A representative thyroid autonomy (hyperthyroidism) is Graves' disease, a common autoimmune disease caused by TSH receptor stimulating antibodies (TRAb) [1]. Hyperthyroidism also results from autonomic functioning adenomas (solitary toxic adenoma or Plummer disease) [1]. Marine-Lenhart syndrome is a rare disorder consisting of both Graves' disease and Plummer disease [2]. There have been various case reports describing Marine-Lenhart syndrome; however, to the best of our knowledge, all previously reported cases except two cases involved a positive TRAb titer [2–4].

In this report, we describe a case of Marine-Lenhart syndrome with a negative TSH receptor antibody titer. Treatment with a fixed, low dose of radioiodine was highly effective for treating hyperthyroidism in this case. We also briefly review and discuss the treatment options for Marine-Lenhart syndrome.

## 2. Case Presentation

A 75-year-old female presented to our hospital with malaise, palpitations, and mild fine tremors. She had felt ambiguous chest discomfort for a long period of time. In addition, she was anxious about her unintentional weight loss. Her past medical history was unremarkable except for untreated hepatitis C virus infection. Mild hyperthyroidism had been detected; however, the accurate diagnosis was not confirmed for at least two years at another hospital. Her family history was negative for endocrinopathy, including thyroid disease. She was currently receiving treatment with amlodipine for hypertension and rivaroxaban for paroxysmal atrial fibrillation. She was thin and appeared tired on general inspection. On a physical examination, she was found to be 156 cm in height and 46 kg in weight (body mass index: 18.9 kg/m^2^). Her blood pressure was 140/80 mmHg and her heart rate was 104 beats per minute. No diffuse goiters were apparent on inspection, and no abnormal masses were palpable in the thyroid gland. She did not exhibit any signs suggestive of Graves' ophthalmopathy, including conjunctival injection, periorbital edema, or proptosis.

The laboratory data were as follows: AST = 46 IU/L (range, 11–28), ALT = 54 IU/L (range, 7–23), ALP = 352 IU/L (range, 115–330), *γ*GTP = 18 IU/L (range, 9–27), TSH = <0.005 *μ*IU/mL (range, 0.2–4.0), free thyroxine = 2.66 ng/dL (range, 1.00–2.00), free triiodothyronine = 11.35 pg/mL (range, 2.30–4.00), and negative thyroid autoantibodies, including negative TRAb, negative anti-thyroglobulin antibodies (TGAb), and negative anti-thyroid peroxidase antibodies (TPOAb). Thyroid autoantibodies were tested twice, with negative results both times. TSH receptor stimulating antibodies (TSAb), as assessed on a bioassay, were also negative.

**Figure 1:**
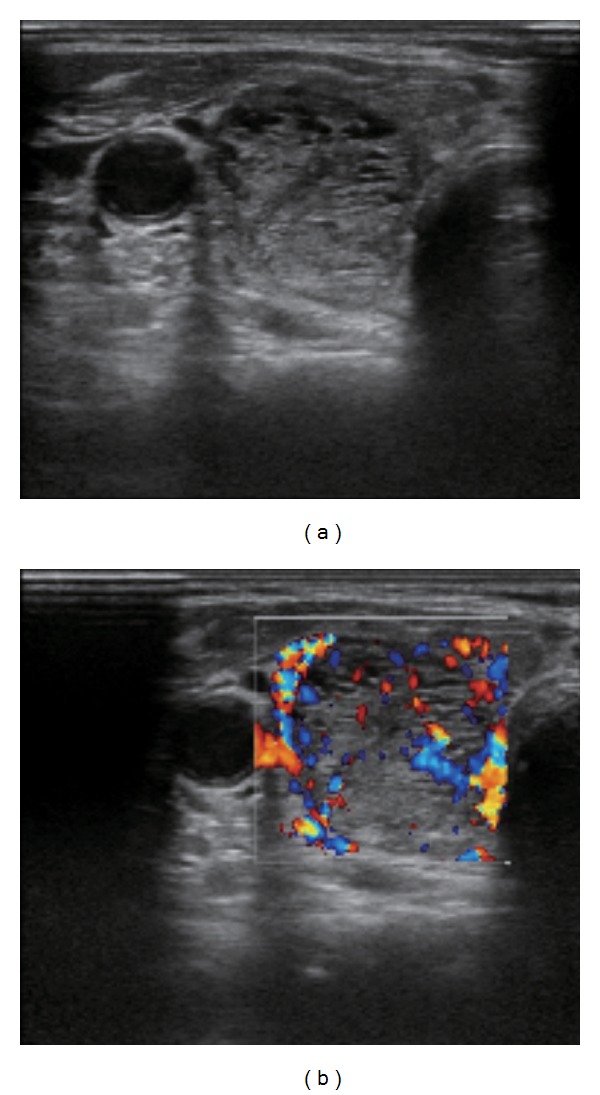
Ultrasonography of the right lobe. A hypoechoic, inhomogeneous mass with a sharp border was detected in the right lobe. The tumor measured 21.5 mm × 19.4 mm × 36.3 mm in diameter. Doppler color flow revealed a high vascularity of the tumor.

**Figure 2:**
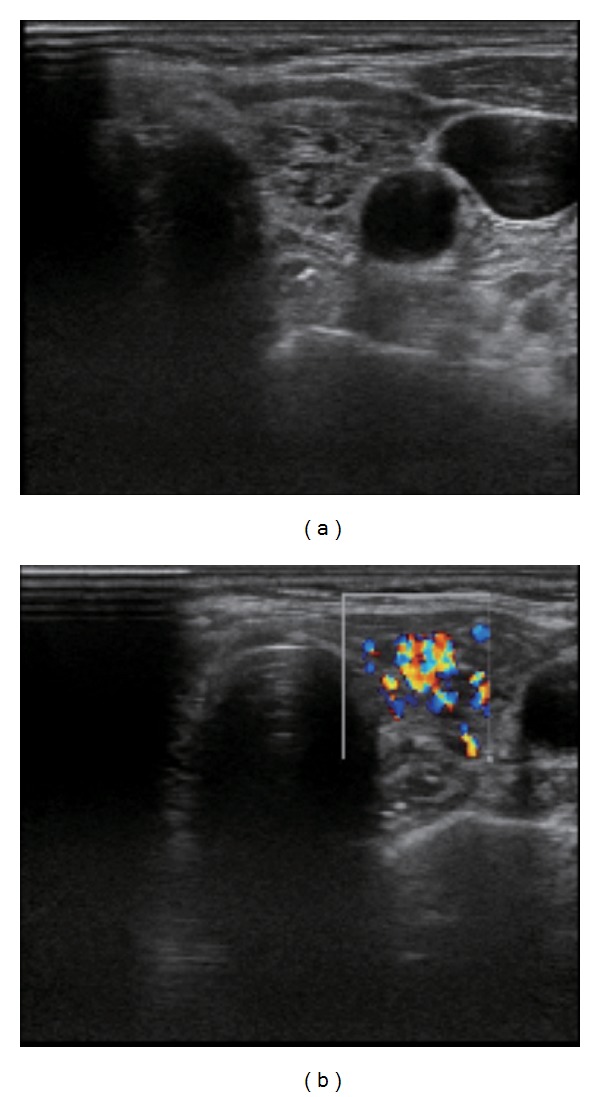
Ultrasonography of the left lobe. The remaining thyroid gland had an inhomogeneous appearance. There were no tumors in the left gland. Doppler color flow revealed a high vascularity in the remaining thyroid gland.

**Figure 3:**
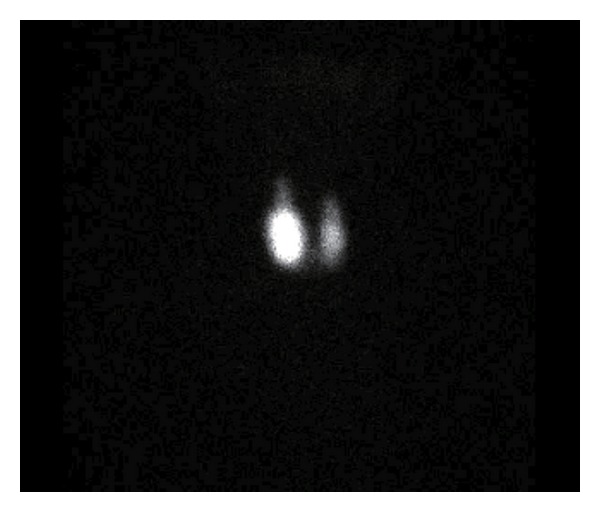
I^123^ scintigraphy of the thyroid gland. There was a strong uptake corresponding to the tumor in the right lobe detected on the ultrasonography. A nonsuppressible uptake was also observed throughout the remaining thyroid gland, thus suggesting underlying Graves' disease. The total uptake percentage of I^123^ for four hours was approximately 24% (range: 4–16%).

Ultrasonography of the thyroid gland revealed a tumor in the right lobe (Figure 1) measuring 21.5 mm × 19.4 mm × 36.3 mm in diameter. The lesion had a relatively low echoic appearance and sharp border. No areas of microcalcification were detected in the tumor, although areas of macrocalcification were present. The remaining thyroid gland had an inhomogeneous, rough texture with a high color Doppler flow (Figure 2). I^123^ scintigraphy was performed following iodine restriction for one week prior to the study, revealing a hot nodule in the lower right thyroid gland corresponding to the tumor detected on ultrasonography (Figure 3). The total uptake percentage of I^123^ for four hours was approximately 24% (range: 4–16%). Surprisingly, there was an increased uptake of radionuclide in the remaining thyroid gland despite the suppressed level of TSH and negative TRAb titer, suggesting underlying Graves' disease.

Thioamides, which are commonly used to treat Graves' disease in Japan, have the well-known side effect of hepatotoxicity [5, 6]. Because the patient displayed elevated liver enzymes due to her untreated hepatitis C, we administered 10 mCi of radioiodine instead of thioamides. After the treatment, her subjective ambiguous chest discomfort, possibly due to paroxysmal atrial fibrillation, was ameliorated. Thyroid function test results on a regular visit conducted one month after the radioiodine treatment were as follows: TSH = <0.005 *μ*IU/mL (range, 0.2–4.0), free thyroxine = 1.40 ng/dL (range, 1.00–2.00), and free triiodothyronine = 4.91 pg/mL (range, 2.30–4.00). Her hyperthyroidism was successfully improved one month after the administration of the radioisotope and no episodes of recurrence have since been observed.

## 3. Discussion

In this case, the results of ultrasonography and I^123^ scintigraphy were compatible with a diagnosis of Marine-Lenhart syndrome. Marine-Lenhart syndrome is a rare disorder, with an incidence of 1~2.7% in patients with Graves' disease [7, 8]. The currently available assay for TSH receptor antibodies has sufficiently high sensitivity and specificity [5]. There is a previous case report of Marine-Lenhart syndrome associated with a negative TRAb titer, positive TPO antibody titer, and positive TG antibody titer [3]. However, in that report, the ultrasonographic study showed no nodularity, as pointed out in a review by Biersack and Biermann [2]. In addition, Chatzopoulos et al. previously reported a case of Marine-Lenhart syndrome associated with a negative TRAb titer [4]. However, in this report, the TSH level was not fully suppressed and a Tc-99m scan showed a homogenously increased uptake throughout the thyroid gland before radioiodine treatment.

Since TRAb was negative in the present report, the diagnosis of Graves' disease was made based on the detection of a homogenous radioiodine uptake in the extranodular thyroid tissue, despite the suppressed level of TSH and the hypervascularity of the extranodular thyroid gland on ultrasonography, as shown in Figure 2. In addition, the high free T3/free T4 ratio observed before treatment suggested the presence of underlying Graves' disease. Although the definitive diagnosis should rely on the pathological findings of the thyroid tissue and we could not obtain histological findings supporting the definite diagnosis of Graves' disease, to the best of our knowledge, we believe that the present report is the first case report of Marine-Lenhart syndrome with a negative TRAb titer showing compatible radiological findings.

The treatment options for Graves' disease include thioamides, radioiodine, and surgery [5]. In Japan and Europe, thioamides are commonly used to treat Graves' disease, while radioiodine is often utilized in the United States [5, 6]. Hepatotoxicity is a well-known side effect of thioamides [5]. Because our patient had a history of untreated hepatitis C and elevated liver enzymes, we treated her with radioiodine in order to avoid hepatotoxicity. In contrast to Graves' disease, Plummer disease is often treated with surgery or radioiodine [5]. Thioamides are seldom used for the treatment for Plummer disease; these drugs are only applied in limited cases [5]. There is an observation that a higher dose of radioiodine is generally required for Marine-Lenhart syndrome due to the development of radioresistance [7]. In another case report, the dose of radioiodine was reported to be 20 mCi for the treatment for Marine-Lenhart syndrome and such a high dose of radioiodine has been reported to lead to the development of postablative hypothyroidism [9]. In contrast to these observations, we successfully treated our patient with a fixed, low dose of 10 mCi of I^131^ in the outpatient setting. Although the utilization of a low dose of I^131^ does not deny the possible occurrence of hypothyroidism in the future, the dose of 10 mCi was sufficiently enough for the treatment of our case. Because Marine-Lenhart syndrome consists of both Graves' disease and Plummer disease, treatment with a relatively low dose of radioiodine is a reasonable first choice for ameliorating both disease etiologies simultaneously.

In conclusion, a negative TSH receptor antibody titer does not necessarily rule out the existence of Graves' disease in patients with Plummer disease. The high free T3/free T4 ratio, ultrasonography, and thyroid scintigraphy are, thus, considered to be helpful for making a diagnosis in such cases. Marine-Lenhart syndrome can, therefore, be successfully managed with a relatively low, fixed dose of radioiodine.
